# Wernicke Encephalopathy Presenting With Hearing Loss and Vision Loss in a Nonalcoholic Patient

**DOI:** 10.7759/cureus.72186

**Published:** 2024-10-23

**Authors:** Amy H Sim, Rui Tang, Thomas J O´Neill, Paisith Piriyawat

**Affiliations:** 1 Neurology, Texas Tech University Health Sciences Center El Paso Paul L. Foster School of Medicine, El Paso, USA; 2 Neuroradiology, Texas Tech University Health Sciences Center El Paso Paul L. Foster School of Medicine, El Paso, USA

**Keywords:** ataxia, binocular diplopia, hearing loss, ophthalmoplegia, vision loss, wernicke encephalopathy

## Abstract

Wernicke encephalopathy is an acute neuropsychiatry syndrome resulting from thiamine deficiency associated with significant morbidity and mortality. We reported a case of a 25-year-old woman with a history of abdomen pain, nausea, vomiting, weight loss, and sore throat who presented with acute neurological symptoms, including binocular diplopia, hearing loss, vision loss, and difficulty ambulating. Examination revealed bilateral vision loss with perception only to light, ophthalmoplegia, hearing loss, gait ataxia, and areflexia. Laboratory work detected multiple vitamin deficiencies. Magnetic resonance imaging (MRI) of the brain showed increased T2 signal in the bilateral medial thalami and periaqueductal areas. Empirical treatment with intravenous thiamine resulted in rapid clinical and radiological resolution.

## Introduction

Wernicke encephalopathy is an acute neurologic complication of thiamine deficiency, associated with high morbidity and mortality requiring emergent treatment. It was first described by Carl Wernicke in 1881 based on clinical observations and autopsy findings of three patients. These patients exhibited acute impairment of consciousness and cognitive function, nystagmus and ophthalmoplegia, and ataxia; the autopsy revealed punctate hemorrhages in the grey matter of the third and fourth ventricles and the aqueduct [1].

Thiamine acts as a cofactor for several enzymes in the tricarboxylic acid (TCA) cycle and pentose phosphate pathways. It is crucial for membrane integrity and cellular osmotic balance. Thiamine deficiency reduces energy production, increases oxidative stress sensitivity, and leads to cellular damage and cytotoxic edema. Astrocytes, capillary endothelial cells, and pericytes are affected, disrupting the blood-brain barrier and causing vasogenic edema [2]. While Wernicke encephalopathy is primarily associated with alcohol abuse, it can also occur due to hyperemesis gravidarum, post-chemotherapy, post-bariatric surgery, chronic total parenteral nutrition [3] or malnutrition from psychiatric conditions [4]. We reported a case of a nonalcoholic patient with an unusual presentation of vision loss, hearing loss, binocular diplopia, ataxia, and symptoms improved with thiamine treatment.

## Case presentation

A 25-year-old woman presented with double vision for 10 days, worsening vision and hearing loss, and difficulty ambulating for five days. She described her vision as initially seeing two images stacked on top of each other and subsequently declining to light perception only. Concomitantly, she experienced progressive hearing loss with persistent tinnitus described as whooshing and clicking sounds. Her husband noted difficulty walking straight where she swayed and eventually could not walk unaided, requiring assistance. Additionally, she also reported bilateral lower extremity numbness and heaviness. Over the past four months, the patient had multiple hospitalizations for nausea, vomiting, difficulty swallowing, and diffuse abdominal pain. She was diagnosed with esophagitis, nonalcoholic steatohepatitis, and gallstone pancreatitis, with a resultant weight loss of 60 pounds. Otherwise, she denied substance use, an eating disorder, or a family history of similar symptoms.

On physical examination, vital signs fluctuated with heart rate and blood pressure. The patient was awake and alert. The visual acuity exam showed only light perception bilaterally. Esotropia on the primary gaze in the left eye and bilateral horizontal gaze-evoked nystagmus were demonstrated. Bilateral hearing loss was also noted. Limb strength was normal. Reflexes were absent. Gait was ataxic and wide-based.

**Figure 1:**
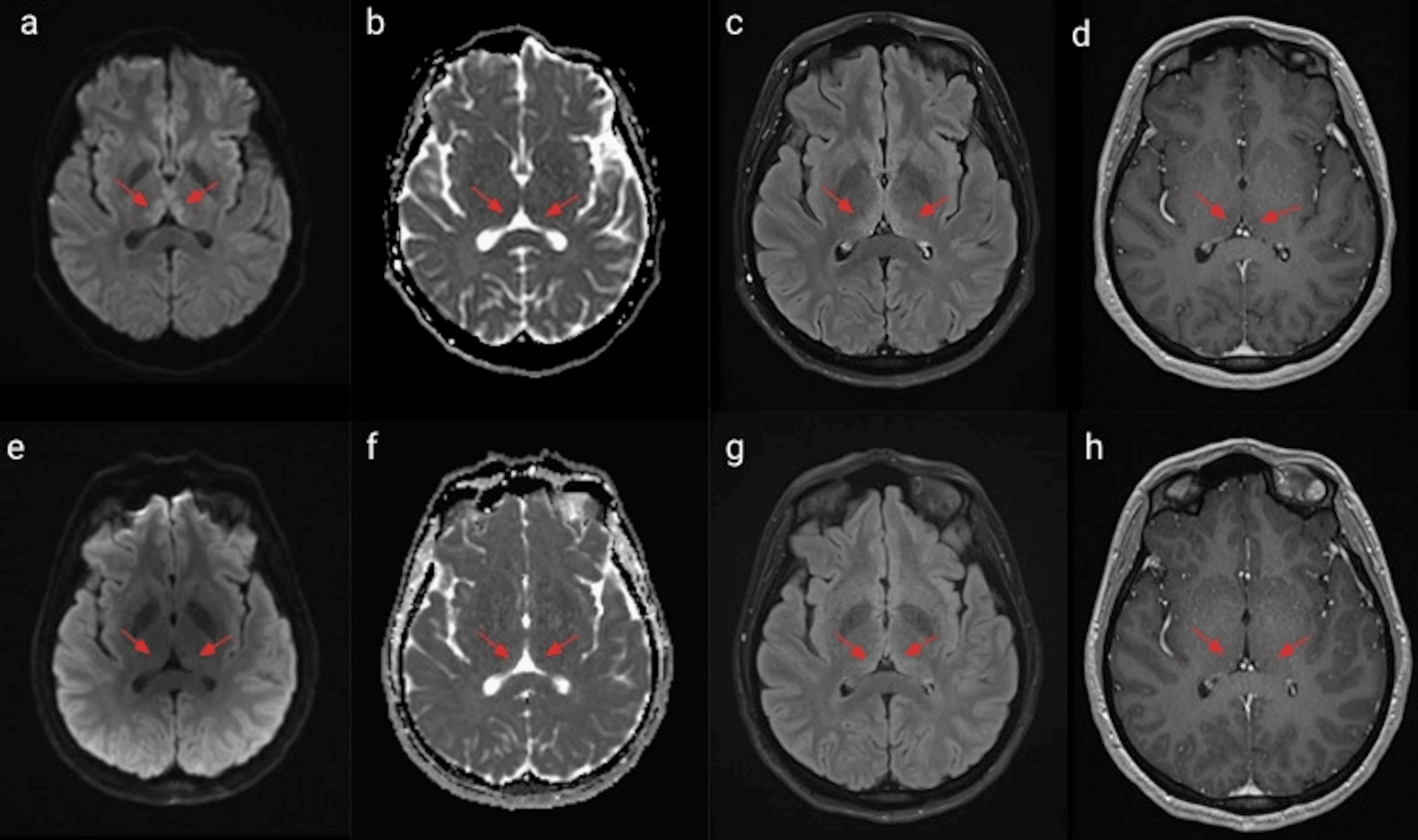
MRI brain comparison of thalami/pulvinar on admission and four months later MRI brain comparison of thalami/pulvinar on admission (the top row) and four months later (the bottom row): diffusion restriction (a, b) and hyperintensity in T2-weighted-Fluid-Attenuated Inversion Recovery (T2 FLAIR) sequence(c). Resolving signal abnormalities with no diffusion restriction (e, f) and minimal residual elevated T2/FLAIR signal (g) in the medial aspect of the thalami, T1-weighted images are normal (d, h).

Intravenous thiamine 500 mg three times daily was promptly initiated based on clinical pictures of ophthalmoplegia, ataxia, hearing loss, vision problems, and a history of gastroenteric issues with weight loss. The patient had significant improvement in her neurologic exam. The following day, she could hear, and the examiner did not need to repeat the questions. Also, she started to see fingers, not just light perception. Clinic improvement, laboratory tests, and images returned later and supported our clinical diagnosis. The laboratory tests revealed multiple vitamin deficiencies, with a thiamine level of less than 7 pg/mL, vitamin K less than 50 pg/mL, vitamin A 20 pg/mL, folate acid 1.86 ng/mL, and vitamin D, 25-OH less than 12.8 ng/mL (Table 1). Brain MRI with gadolinium showed increased T2 signal in the medial thalamic areas bilaterally and periaqueductal region (Figure 1), supporting the diagnosis. Electromyography (EMG) and nerve conduction studies (NCS) were normal.

**Table 1:** Laboratory Tests Laboratory tests revealed multiple vitamin deficiencies, with a thiamine level of less than 7 pg/mL, vitamin K less than 50 pg/mL, vitamin A 20 pg/mL, folate acid 1.86 ng/mL, and vitamin D, 25-OH less than 12.8ng/mL. Methylmalonic acid and Immunoglobulin (Ig)G subclass are in the normal range, GQ1B Ab IgG, GM1 Ab IgG, GM1 Ab IgM, and HIV 1/2 were negative.

Test	Value	Reference
Vitamin A	20	38-98mcg/dL
Thiamin	<7	8-30nmol/L
Folate acid	1.86	2.76-20.0ng/mL
Vitamin B12	417	239-931pg/mL
Vitamin D 25-OH	<12.8	30-100
Vitamin K	<50	130-1500
Methylmalonic acid	74	87-318nmol/L
GQ1 B Ab IgG	<1:100	<1:100
GM1 Ab IgG	<1:800	<1:800
GM-1 Ab IgM	<1:800	<1:800
Immunoglobulin G subclass	44.4	4-86mg/dL
HIV1/2	negative	N/A

**Figure 2:**
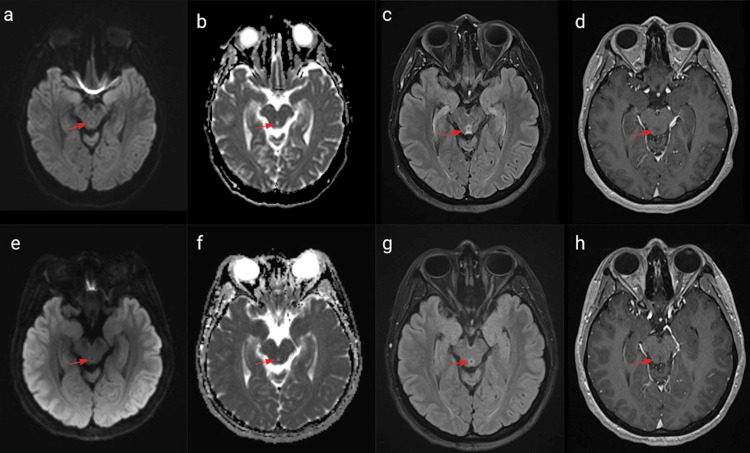
MRI brain comparison of the periaqueductal region on admission and four months later MRI brain comparison of the periaqueductal region on admission (the top row) and four months later (the bottom row): diffusion restriction (a, b) and hyperintensity in T2-weighted-Fluid-Attenuated Inversion Recovery (T2 FLAIR) sequence(c). Resolving signal abnormalities with minimal diffusion restriction (e, f) and trace residual T2/FLAIR signal (g) on the periaqueductal region. T1-weighted images are normal (d, h).

After intravenous thiamine three times daily for five days with other vitamin supplements, the patient was discharged with oral thiamine 250 mg daily, ergocalciferol 50000 intern-unit, once caps once a week, multivitamin with minerals one tab daily, folic acid 1 mg daily and continued physical therapy and follow the diet dietician recommended. In the four-month follow-up, vision and hearing had recovered, although she remained sensitive to loud noise and bright light. She could walk with caution or with assistive devices. Follow-up imaging indicated improvement in thalamic signal abnormalities (Figure 1) and resolving abnormalities in the periaqueductal region (Figure 2) with no associated enhancement on contrast-enhanced T1-weighted images. The patient was also referred to see a neuro-ophthalmologist. Unfortunately, the patient lost follow-up after the first visit to the clinic. However, we considered following up on the patient’s symptoms and thiamine level.

## Discussion

While Wernicke encephalopathy is straightforward when a patient presents with the classic triad of encephalopathy, oculomotor dysfunction, and gait ataxia in chronic heavy alcohol use, this accounts for only one-third of cases [5]. Diagnosis may become challenging due to various presentations and the absence of alcohol dependence. In addition, missing the clinical symptoms and signs is very common during the clinical approach. Back in 1986, 19% of patients who were diagnosed with Wernicke-Korsakoff at necropsy had no documented clinical signs, and 80% were not diagnosed during their life [6].

In clinical practice, obtaining comprehensive patient information can be challenging. In this case, the patient's frustration and difficulty responding to questions, even with loud and vigorous stimulation, were attributed to hearing loss, possibly confounding by inattention. Encephalopathy in Wernicke encephalopathy includes symptoms such as inattentiveness, disorientation, indifference, apathy, memory loss, learning impairment, dizziness, delirium, lethargy, or coma. Thiamine deficiency can cause dysautonomia via impairing mitochondrial function, leading to autonomic nervous system dysfunction that can explain the patient's fluctuating vital signs. According to the literature, hypotension and hypothermia are more prominent [7].

The patient exhibited difficulty performing finger-to-nose and heel-to-shin tasks, with a slow, short-spaced, wide-based gait requiring assistance. The reported numbness and heaviness in bilateral lower extremities, combined with hearing loss, suggest gait ataxia due to a combination of peripheral neuropathy, vestibular disturbance, and cerebellar dysfunction.

Nystagmus, typically horizontal and provoked by lateral gaze, is the most common manifestation, reflecting lesions in the oculomotor, abducens, and vestibular nuclei [8]. Interestingly, most cases of vision loss in Wernicke encephalopathy occur in nonalcoholic patients, such as those with Crohn’s disease, ulcerative colitis, hyperemesis gravidarum, leukemia, or COVID-19-induced thiamine deficiency. Vision loss can precede other symptoms and imaging abnormalities [9]. Pathological findings include peripapillary nerve fiber layer thickening, telangiectasia, and retinal hemorrhages due to mitochondrial dysfunction in retinal ganglion cells and capillaries [10].

Hearing loss in Wernicke encephalopathy is increasingly recognized. Approximately eight cases have been reported, with MRI showing bilateral thalamic hyperintensities in six cases, bilateral inferior colliculi hypodensity in one case, and no MRI findings in another [11]. Similar to vision loss, hearing loss is predominantly reported in nonalcoholic patients. It is a significant symptom, sometimes developing months after other symptoms have resolved [12]. Patients may experience hearing loss alone or with tinnitus described as whooshing, clicking, or experiencing palinacousis-an auditory illusion where external sounds echo internally after cessation [13].

Despite limited history, physical examination, and atypical symptom presentation, this patient did not present with hypokalemia, hypomagnesemia, or elevated international normalized ratio suggestive of malnutrition. Initially, this posed a diagnostic dilemma, with a broad differential including Guillain-Barre syndrome variants with dysautonomia, Miller Fisher Syndrome, thiamine deficiency causing wet and dry beriberi, and Susac’s syndrome.

According to European Federation of the Neurological Societies (EFNS) guidelines, clinical diagnosis of Wernicke encephalopathy in alcoholics requires meeting two of the following four Caine criteria: dietary deficiency, eye signs, cerebellar dysfunction, and altered mental status or mild memory impairment [14]. It is crucial to recognize different presentations between alcoholics and non-alcoholics, although a clinical diagnosis can also follow the Caine criteria. While MRI is highly specific (93%), its sensitivity is lower (53%) [15]. Thiamine has an excellent safety profile [7]. If clinical suspicion is high, parenteral thiamine should be administered immediately after blood sampling for total thiamine levels, even before MRI.

## Conclusions

Currently, the diagnosis of Wernicke encephalopathy relies on careful clinical observation. Our case, featuring an uncommon combination of hearing loss, vision loss, and gait ataxia, and dramatic improvement following immediate intravenous thiamine treatment, contributes significantly to the literature on Wernicke encephalopathy in nonalcoholic populations. It underscores the importance of extended history-taking and thorough assessment of these patients.
